# Health problems of people with intellectual disabilities in general practice: dynamic cohort study between 2012 and 2021 with Dutch routine care data

**DOI:** 10.3399/BJGP.2025.0084

**Published:** 2025-12-02

**Authors:** Marloes Heutmekers, Bianca Schalk, Annemarie Uijen, Jenneken Naaldenberg, Geraline Leusink, Maarten Cuypers

**Affiliations:** 1 Radboud University Medical Center, Department of Primary and Community Care, Nijmegen, the Netherlands; 2 Academic Collaborative Intellectual Disability and Health, Sterker op Eigen Benen, Nijmegen, the Netherlands

**Keywords:** primary health care, general practice, intellectual disability, cohort studies, depression, prescriptions

## Abstract

**Background:**

Despite advancements in health care, patients with intellectual disabilities (ID) in many countries continue to face barriers in accessing and utilising primary care. Implementation of improvements in accessibility and quality of care requires up-to-date and accurate insights into their health problems.

**Aim:**

To investigate health problems in patients with ID in GP care compared with matched patients without ID.

**Design and setting:**

A retrospective dynamic cohort study undertaken using data from >80 Dutch general practices.

**Method:**

All adult patients with indicators of ID, registered at any participating general practice for a minimum of 1 year between 2012 and 2021, were included, and individually matched (1:5) with persons without ID. Patients’ characteristics, encounters, symptoms, diagnoses, and prescribed medication were retrieved.

**Results:**

Patients with ID had 2.2 times more contacts with their GP than patients without ID, presented with a broader range of symptoms and diagnoses across various body systems, and were more frequently prescribed medication. The largest relative difference was seen for depression, which was nearly twice as common in patients with ID compared with those without.

**Conclusion:**

The health problems and prescription patterns of people with ID in general practice remain distinct from those without ID but largely mirror findings from two decades ago. These patterns still fit well within the scope of general practice, yet underscore the continuing need for GPs to recognise these differences and adapt their care to address the specific needs of their patients with ID.

## How this fits in

People with intellectual disabilities (ID) present unique challenges for GPs owing to their generally higher morbidity rates and distinct care needs compared with the general population. Accurate insights into the symptoms and diagnoses common among patients with ID and how they differ from those without ID would aid GPs in tailoring their care; however, there is limited complete and contemporary insight into such patterns. This study addresses this gap by analysing routine GP care data from a large-scale investigation with 10-year follow-up, comparing health symptoms and diagnoses in patients with ID with matched patients without ID. The present findings show the most commonly occurring health problems among patients with ID fit within the GP setting, yet the frequency of contacts and number of co-occurring conditions underscore their vulnerability and the importance of ongoing health monitoring and collaboration with specialised ID care to ensure optimally tailored care.

## Introduction

People with intellectual disabilities (ID) present a heterogeneous group of conditions characterised by limitations in cognitive functioning and adaptive behaviour that manifest during the developmental period.^1^ Approximately 1.5% of the population has an ID.^2–4^ Having an ID coincides with a higher prevalence of chronic illness and several other health problems,^5,6^ and consequently higher healthcare demands^7,8^ and different health needs than the general population.^9^ This results in an elevated risk of premature death, mortality rates three times higher than the general population, and a greater proportion of deaths from causes considered avoidable.^10–15^

Health care for people with ID is organised differently across countries.^16^ In the Netherlands, GPs are healthcare gatekeepers, as their assessment and referral are required to access hospital and medical specialist care. GPs typically serve as first point of contact for people with ID and their families.^17^ However, in primary care, many challenges persist in the provision of accessible and good-quality care for them.^18–21^ To inform GPs with the different health needs of people with ID, van Schrojenstein Lantman-De Valk *et al* two decades ago investigated the main differences in health problems between patients with and without ID in Dutch general practice.^7,22^ Important findings were that patients with ID were 2.5 times more likely to have health problems — mostly neurological or psychological — compared with those without ID.

Both practice and research have, however, evolved since van Schrojenstein Lantman-De Valk *et al*'s seminal work.^7^ There are now improved methods to identify ID, improved longevity of the population,^23^ electronic medical records for accuracy and standardisation, further development of (evidence-based) guidelines and care standards in general practice, and — of particular relevance to the Netherlands — the introduction of the ID physician as medical specialist in 2000 and the introduction of a reformed Long-term Care Act in 2015. While these factors have impacted health and health care of people with ID in Dutch primary care, in similar fashion to international evolutions in health care for people with ID,^16^ there has been limited research to update those earlier findings. This study therefore adopted a similar approach to compare GP care between patients with and without ID, applying concurrent methods to identify patients with ID.

## Method

### Setting, design, and data source

This retrospective dynamic cohort study utilises data from the GP database of the Department of Primary and Community Care at the Radboud University Medical Center, the Netherlands, which contains patients’ electronic health records for >80 general practices and provides data on demographics, consultations, physical measurements (laboratory results, blood pressure, and so on), prescribed medications, referrals, symptoms, and diagnoses. This large database is proven to provide representative insight into population-level health outcomes.^24–26^ Patients may decline inclusion of their individual data in the database (opt-out procedure).

### Selection and matching

**Figure 1.**
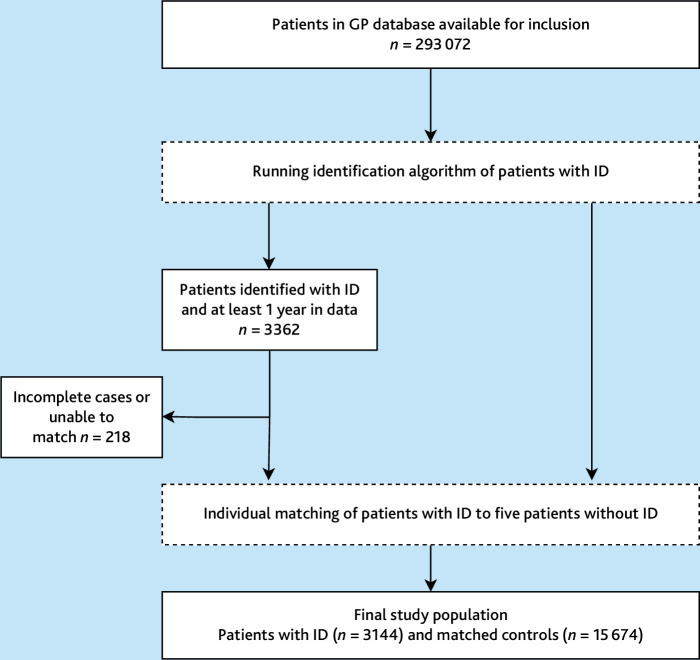
Inclusion of 3144 patients with ID and 15 674 matched controls. ID = intellectual disabilities.

All adult patients were eligible for enrolment in this study if registered at any of the participating general practices for a minimum of 1 year between 2012 and 2021 (Figure 1). People with ID were identified using an algorithm combining a selection of relevant coding and text entries in the medical record indicative for ID.^27^ Next, patients with ID were individually matched (1:5) with persons without ID based on age (year of birth±1 year), sex, GP practice, and same registration period. As patients can join or withdraw from the GP practice at any time, cohort time could be between 1 year and 10 years.

### Data and outcomes

Patient characteristics (sex and year of birth), patient encounters (all types of consultation observed during cohort time), symptoms and diagnoses, and prescribed medication were retrieved. All symptom or diagnosis episodes starting within the cohort time and all diagnoses of chronic illness regardless of the date of diagnosis were included.

Symptoms and diagnoses are coded according to the International Classification of Primary Care (ICPC), either version 1 or 2.^28^ In addition to symptoms and diagnoses being reported by the three-position ICPC code, the 15 body systems, general unspecified health problems, and social problems were reported by the first letter of the ICPC classification.^29,30^ The medication prescribed during cohort time was encoded using the seven-element Anatomical Therapeutic Chemical (ATC) classification.^31^ Following this classification, the terms 'psycholeptics' (sedatives or inhibitors; N05) and 'psychoanaleptics' (stimulants; N06) were used separately, rather than the broader term 'psychotropics', which is more commonly used in general practice.

### Statistical analyses

Patient and GP practice characteristics were presented as descriptive statistics. Means were presented with standard deviation (SD), median with interquartile range (IQR), and frequencies (*n*) with percentage (%). Patients’ age in 2021 was calculated using birth year. The mean number of consultations per year was calculated by dividing the total number of consultations by the cohort time in years. Each first registration of specific symptoms or diagnoses during cohort time was used, except for chronic diseases. The most commonly reported symptoms and diagnoses in persons with ID were reported. A similar method was used for determining the occurrence of health problems by the 17 ICPC chapters being the 15 body systems, general unspecified health problems, and social problems. Comparisons with the general population were made by calculating relative risks (RRs) and their 95% confidence intervals (CIs).^32^ The ICPC codes P85 (mental retardation) and A90.01 (Down syndrome) were excluded as outcomes as they served as indicator variables for ID as well.

As the study period included the COVID-19 pandemic, a sensitivity analysis was conducted describing the commonly recorded symptoms and diagnoses during (2020–2021) and pre-pandemic (2012–2019) in order to test any disrupting effect of the pandemic. SPSS Statistics (version 29.0) was used for all analyses. Outcomes are reported in accordance with the REporting of studies Conducted using Observational Routinely collected Data (RECORD) statement.

## Results

The 83 general practices served a total of 289 710 patients who met the inclusion criteria. ID prevalence in this patient population was 1.2% (*n* = 3362/289 710 patients). Practices had a median of 29 patients with ID (IQR 17–47). After matching with non-ID controls, the cohort consisted of 3144 patients with ID and 15 674 matched controls. Matching was incomplete for 18 patients with ID, who were included with between one to four matched controls. Figure 1 shows the inclusion process.

The total study population (Table 1) was relatively young, with about 77% aged <55 years, and 53.8% male. Average cohort time was 7.7 years (SD 3.1), with more than half of participants registered for the full 10-year study period. Most patients had at least one contact with their GP during the cohort time, with 99.2% (*n* = 2825/2849) in the ID group and 96.5% (*n* = 13 714/14 206) among the matched controls. Patients with ID (6.5 per year, IQR 3.1–12.2) had on average 2.2 times more contacts per year when compared with the matched controls (3.0 per year, IQR 1.4–5.7) for all types of contacts combined. Patients with ID were twice as likely to have an average of ≥5 contacts per year (60.2% versus 29.6%).

**Table 1.** Patient characteristics (*N* = 18 818)

	Patients with ID (*n* = 3144)		Matched controls without ID (*n* = 15 674)	
**Category^a^**	***n***	**%**	***n***	**%**
Sex				
Male	1693	53.8	8432	53.8
Female	1451	46.2	7242	46.2
Age, years^b^				
<25	646	20.5	3231	20.6
25–34	839	26.7	4166	26.6
35–44	500	15.9	2507	16.0
45–54	435	13.8	2172	13.9
55–64	379	12.1	1885	12.0
65–74	187	5.9	935	6.0
75–84	112	3.6	560	3.6
≥85	46	1.5	218	1.4
Cohort time, years				
≤1	221	7.0	1100	7.0
2–3	399	12.7	1987	12.7
4–5	287	9.1	1433	9.1
6–7	227	7.2	1134	7.2
8–9	319	10.1	1582	10.1
10	1691	53.8	8438	53.8
Average number of contacts per year				
No information	295		1468	
None	24	0.8	492	3.5
≤1	407	14.3	4554	32.1
2–5	701	24.6	4956	34.9
6–10	782	27.4	2888	20.3
11–20	627	22.0	1120	7.9
≥21	308	10.8	196	1.4

^a^Sex, age, and cohort time were part of the matching criteria and therefore almost the same for both groups. ^b^Age is calculated for 2021 (last year of follow-up). ID = intellectual disabilities.

The commonest health problems of patients with ID are presented in Table 2, separately for diagnoses and complaints. The three most commonly recorded diagnoses were as follows: ‘no disease (A97)’ (50.5% of patients with ID had an encounter resulting in this diagnosis); ‘upper respiratory infection acute (R74)’ (26.0%); and ‘dermatophytosis (S74)’ (21.1%). The three commonest complaints and symptoms were as follows: ‘weakness/tiredness general (A04)’ (23.4%); ‘foot/toe symptom/complaint (L17)’ (20.7%); and ‘cough (R05)’ (20.5%). The largest relative difference was found for ‘depressive disorder (P76)’, with people with ID being almost twice as likely to have had this diagnosis (RR 1.9, 95% CI = 1.7 to 2.1) at least once during follow-up.

**Table 2.** Ten most commonly presented health problem and relative risk of patients with intellectual disabilities in general practice in diagnoses and complaints episodes compared with matched controls

		Patients with ID (*n* = 3144)		Matched controls without ID (*n* = 15 674)		
**Number**	**Diagnoses and conditions**	***n***	**%**	***n***	**%**	**RR (95% CI)**
1	No disease (A97)	1587	50.5	5006	31.9	1.58 (1.49 to 1.67)
2	Upper respiratory infection acute (R74)	819	26.0	2912	18.6	1.40 (1.30 to 1.52)
3	Dermatophytosis (S74)	663	21.1	2262	14.4	1.46 (1.34 to 1.59)
4	Dermatitis contact/allergic (S88)	657	20.9	2972	19.0	1.10 (1.01 to 1.20)
5	Excessive ear wax (H81)	605	19.2	2107	13.4	1.43 (1.31 to 1.57)
6	Injury musculoskeletal NOS (L81)	566	18.0	2079	13.3	1.36 (1.24 to 1.49)
7	Allergic rhinitis (R97)	543	17.3	2806	17.9	0.96 (0.88 to 1.06)
8	Cystitis/urinary infection other (U71)	542	17.2	1916	12.2	1.41 (1.28 to 1.55)
9	Musculoskeletal disease, other (L99)	509	16.2	2275	14.5	1.12 (1.01 to 1.23)
10	Depressive disorder (P76)	470	14.9	1261	8.0	1.86 (1.67 to 2.07)
	**Complaints and symptoms**					
1	Weakness/tiredness general (A04)	736	23.4	2551	16.3	1.43 (1.33 to 1.56)
2	Foot/toe symptom/complaint (L17)	650	20.7	2277	14.5	1.42 (1.30 to 1.55)
3	Cough (R05)	646	20.5	2499	15.9	1.29 (1.18 to 1.41)
4	Chest symptom/complaint (L04)	573	18.2	2277	14.5	1.25 (1.14 to 1.37)
5	Laceration/cut (S18)	498	15.8	1961	12.5	1.27 (1.15 to 1.40)
6	Back symptom/complaint (L02)	497	15.8	1804	11.5	1.37 (1.24 to 1.52)
7	Knee symptom/complaint (L15)	486	15.5	2078	13.3	1.17 (1.06 to 1.29)
8	Abdominal pain localised other (D06)	470	14.9	1636	10.4	1.43 (1.29 to 1.59)
9	Hand/finger symptom/complaint (L12)	470	14.9	1702	10.9	1.38 (1.24 to 1.52)
10	Shoulder symptom/complaint (L08)	459	14.6	1891	12.1	1.21 (1.09 to 1.34)

ID = intellectual disabilities. NOS = not otherwise specified. RR = risk ratio.

**Figure 2.**
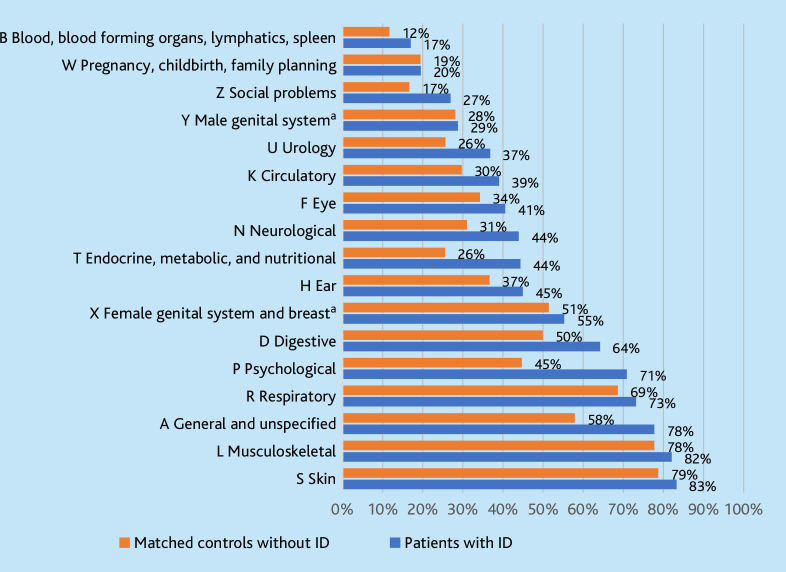
Percentage of patients with a health problem per body tract, for patients with ID compared with the matched controls. Letters refer to the first letter of the ICPC classification. ^a^Calculated for female and male only. ID = intellectual disabilities.

Figure 2 shows the percentages of patients with at least one recorded health problem for each of the 15 body systems, general unspecified health problems, and social problems. For all chapters, the percentage of patients with ID having a history of health problems in the respective chapter is either similar to, or higher than, that of their matched peers. Highest differences were seen within the chapters ‘psychological (P)’ (70.9% versus 44.7%), ‘general and unspecified (A)’ (77.7% versus 57.9%), ‘endocrine, metabolic, and nutritional (T)’ (44.4% versus 25.6%), ‘digestive (D)’ (64.2% versus 49.9%), ‘neurological (N)’ (43.9% versus 31.0%), ‘urology (U)’ (36.8% versus 25.6%), and ‘social problems (Z)’ (26.9% versus 16.6%). Occurrences of codes related to ‘pregnancy, childbirth, family planning (W)’ (19.5% versus 19.4%), ‘male genital system (Y)’ (28.8% versus 28.1%), and ‘female genital system and breast (X)’ (55.3% versus 51.4%) were comparable for both groups.

Most patients both with (97.1%) and without (95.5%) ID were prescribed at least one type of medication during the study period. Table 3 presents the primary ATC subgroups for which patients received prescriptions. Antibacterials for systemic use (J01) were most common, with 62.8% of patients with ID having received at least one prescription from this category, compared with 54.4% among matched controls. Relative differences were largest for ATC subgroups ‘psycholeptics (N05)’ (47.2% versus 21.1%) and ‘psychoanaleptics (N06)’ (33.7% versus 15.4%). Only for ‘vaccines (J07)’ prescription rates were slightly lower in patients with ID (60.8%) than among matched controls (69.0%).

**Table 3.** Ten most common prescription groups with ATC code of patients with intellectual disabilities compared with the matched controls

		Patients with ID(*n* = 3054)		Matched controls without ID (*n* = 14 965)	
**Number**	**Prescription group**	***n***	**%**	***n***	**%**
1	Antibacterials for systemic use (J01)	1919	62.8	8147	54.4
2	Vaccines (J07)	1856	60.8	10 324	69.0
3	Anti-inflammatory and antirheumatic products (M01)	1727	56.5	7613	50.9
4	Psycholeptics (N05)	1443	47.2	3158	21.1
5	Drugs for acid-related disorders (A02)	1328	43.5	4612	30.8
6	Corticosteroids, dermatological preparations (D07)	1218	39.9	5180	34.6
7	Drugs for constipation (A06)	1132	37.1	3538	23.6
8	Antifungals for dermatological use (D01)	1091	35.7	3798	25.4
9	Analgesics (N02)	1080	35.4	3916	26.2
10	Psychoanaleptics (N06)	1029	33.7	2312	15.4

ATC = Anatomical Therapeutic Chemical. ID = intellectual disabilities.

Sensitivity analysis showed an increase in encounters for ‘Fear of other diseases’ and ‘Prevention’ during the COVID-19 pandemic. However, the commonest health problems were the same during the pandemic as before (see Supplementary Table S1).

## Discussion

### Summary

This study has provided updated insights on the differences between patients with and without ID in Dutch general practice through analysis of GP electronic health records in a linked regional GP database. Results showed that between 2012 and 2021, patients with ID contacted their GP twice as often than patients without ID. Most common symptoms and complaints were either generic in nature (that is, no disease, general weakness/tiredness, and cough), infection-related (that is, acute upper respiratory infection and dermatophytosis), or orthopaedical (that is, foot/toe complaints). Patients with ID more often presented psychological health problems, with depression being twice as common, as were psychoanaleptic medication prescriptions.

### Strengths and limitations

This study utilised high-quality routine care data collected in a large regional GP database.^33^ This allowed the authors to retrieve data for this large patient sample over a long time period and apply strict selection criteria for case definition. In addition, the enhanced algorithm included textual cues that helped to identify more patients with ID beyond solely relying on diagnostic codes, which is known for underreporting.^27^ This method yielded an ID prevalence of 1.2%, in line with international literature.^4^

By individually matching patients with ID to non-ID controls of the same age and sex, the differences in both populations’ sex and age profile have been addressed. However, the matched controls are not fully representative of the general primary care population, as these factors influence health outcomes, disease prevalence, and management. For example, finding of an average of 3.0 GP contacts per year among matched controls is much lower than the national average of 5.2 and might be explained by the baseline differences, with the age group that typically generates the most contacts in general practice (older people) underrepresented in this study.^34^

A limitation of using routine care data for research purposes is the challenge of interpreting findings. For instance, the code for ‘no disease’ is used only sparingly to classify a patient’s encounter with literally no underlying health problem and is often used for administrative tasks, preventive encounters, and vaccinations. It does not represent actual clinical presentations, but it does reflect health service use and workload as experienced in general care. The use and understanding of the ‘no disease’ episode has been reported and discussed elsewhere.^7,35,36^

### Comparison with existing literature

Comparable with findings from two decades ago, patients with ID were found to be twice as likely than patients without ID to contact their GP.^7^ The most commonly presented health problems and prescriptions are also to a large extent similar to two decades ago. Structural research on consultation patterns in primary care and national health monitoring programmes could provide insight into possible trends and changes in health needs and could drive quality improvement. While the present study does not explain why care needs remain comparable with those observed two decades ago, despite improvements in healthcare and policy, it does confirm that the health of people with ID remains an urgent topic. Addressing this issue requires sustained insights from high-quality data and international exchange of best practices to support GPs in meeting the needs of patients with ID.^16^

The most prominent difference in observed health problems is the absence of diabetes mellitus in the commonly presented diagnoses in this study. Diabetes care in Dutch general practice has considerably improved with the introduction of the practice assistant in 1999 alongside multidisciplinary care standards.^37^ This particularly impacted care for people with ID, who have higher prevalence rates and generally a younger onset of diabetes mellitus, ensuring better recognition of its complexity and earlier referral to specialist care.^6^ In addition, epilepsy diagnoses, and prescriptions for anti-epileptics and anticonvulsants are in similar fashion not part of the most common findings in this study. Epilepsy management has improved over the years with more coordination between hospitals and tertiary hospitals for complex epilepsy in patients with ID. Anti-epileptics and anticonvulsants may have been discontinued over the years, as diagnoses and treatment of epilepsy in ID have improved, and, additionally, awareness of the limited efficacy in challenging behaviour in ID has increased.^38–40^ Finding similar occurrences of diagnoses and complaints on reproductive health between women with and without ID is in line with earlier research in this area in Dutch general practice.^41^

The finding that depression was recorded almost twice as often among patients with ID compared with those without aligns with previous research. Although estimates on the prevalence of depression vary, its impact may be greater owing to factors such as underrecognition of symptoms and treatments that are insufficiently tailored to the co-occurrence of ID.^42,43^ When specialised mental health services are unable to provide appropriate or effective care, the responsibility for management may increasingly fall to GPs, highlighting the importance of equipping primary care with the tools and knowledge needed to support this group.

### Implications for research and practice

The present study findings have shown that contact frequency and problem diversity distinguish the ID population from the general patient population in general practice. Despite being a distinct patient population, the presented health problems are suitable within GP care. However, mental health problems in combination with an ID can prove challenging in general practice. Frequent prescriptions of psycholeptics (47.2%) and psychoanaleptics (33.7%) were observed for people with ID, those psychotropics do not typically fall within the scope of GP care. These medications are often prescribed for challenging behaviour in people with ID without diagnosed mental health problems.^44,45^ The present study findings have shown that almost three-quarters (71%) of the ID group had at least one psychological complaint or diagnoses within the study period, compared with less than half (45%) among the matched controls. Further investigation is required into the relation between complaints, diagnoses, and prescribed medication to better understand the potential risks for underdiagnosis of mental health problems and overtreatment with neuropsychiatric medication.^46^ Additional training and possibilities for consultation with, or referral to, specialised ID care might be helpful.

While the 2015 legislative reforms and earlier introduction of the ID physician in the Dutch healthcare system in 2000 were reason to repeat a similar study from before this period, their specific impact could not be assessed in the current study design, and was therefore not analysed as such.^7^ And even though the current data did not provide information on whether an ID physician was involved in individual cases, the availability of this medical specialism in the Netherlands may support earlier recognition and improved coordination of care for people with ID.^46^ In other countries without such a specialist role, GPs may face even greater challenges, which underscores the relevance of the present findings in supporting GPs to better understand and address the specific health needs of this patient group. Nevertheless, initiatives such as annual health checks or the STOMP (STopping Over Medication of People with a learning disability, autism, or both) programme in the UK illustrate proactive approaches to tailored care and reducing inappropriate prescribing.^47,48^

As the study period included the COVID-19 pandemic, a sensitivity analysis was conducted comparing commonly recorded symptoms and diagnoses during the pandemic years (2020–2021) with the pre-pandemic period (2012–2019), to assess whether the pandemic disrupted overall patterns in the study findings. Although previous studies have reported increased psychotropic use during the pandemic among people with ID, the present dataset was not designed to investigate prescribing trends in detail, such as initiation, dosage, or treatment changes.^49,50^ Nonetheless, for pandemic preparedness, it would be valuable for future research to explore temporal trends in GP usage, psychotropic prescribing, and mental health management, to better understand the impact of acute public health crises on people with ID.

In conclusion, the present findings confirm that patients with ID are still more frequent GP care users than patients without ID. With more than twice the number of contacts of patients without ID, this patient group is of significant relevance for a tailored GP approach. Relevant insights into their health problems and needs are crucial to optimally serve this population who depend on their GP as the first point of contact. The health problems and prescription patterns observed largely mirror those from two decades ago, with the notable absence of diabetes and epilepsy from current findings. This may reflect changes in diagnostic and treatment guidelines, and improvements in access to specialised care for those conditions for patients with ID. Furthermore, awareness has been raised regarding accurate diagnosis and prescription routines for mental health conditions in relation to ID in primary care, as current practice may be suboptimal.

Overall, the present findings underscore the vulnerability of people with ID and the importance of ongoing health monitoring and collaboration with specialised ID care in primary care to ensure optimally tailored care.

## Data

The data that support the findings of this study are not openly available as they contain individual-level medical records. Aggregated results and infomation about data access are available from the corresponding author on reasonable request. Data are located in controlled access data storage at Radboud University Medical Center.
